# Chitosan Based Aerogels with Low Shrinkage by Chemical Cross-Linking and Supramolecular Interaction

**DOI:** 10.3390/gels8020131

**Published:** 2022-02-18

**Authors:** Sizhao Zhang, Qi Xiao, Yunyun Xiao, Zhengquan Li, Shixian Xiong, Feng Ding, Junpeng He

**Affiliations:** 1 Polymer Aerogels Research Center, Jiangxi University of Science and Technology, Ganzhou 341000, China; xqshawn@163.com (Q.X.); qqzhengquan@163.com (Z.L.); s.xiong@jxust.edu.cn (S.X.); dingfengjl@163.com (F.D.); hjp15170757014@163.com (J.H.); 2 Postdoctoral Research Station on Mechanics, College of Aerospace Science and Engineering, National University of Defense Technology, Changsha 410073, China

**Keywords:** chitosan aerogels, shrinkage, nanofibrillar cellulose, mechanical property, thermal insulation

## Abstract

Chitosan (CTS) aerogel is a new type of functional material that could be possibly applied in the thermal insulation field, especially in energy-saving buildings. However, the inhibition method for the very big shrinkage of CTS aerogels from the final gel to the aerogel is challenging, causing great difficulty in achieving a near-net shape of CTS aerogels. Here, this study explored a facile strategy for restraining CTS-based aerogels’ inherent shrinkage depending on the chemical crosslinking and the interpenetrated supramolecular interaction by introducing nanofibrillar cellulose (NFC) and polyvinyl alcohol (PVA) chains. The effects of different aspect ratios of NFC on the CTS-based aerogels were systematically analyzed. The results showed that the optimal aspect ratio for NFC introduction was 37.5 from the comprehensive property perspective. CTS/PVA/NFC hybrid aerogels with the aspect ratio of 37.5 for NFC gained a superior thermal conductivity of 0.0224 W/m·K at ambient atmosphere (the cold surface temperature was only 33.46 °C, despite contacting the hot surface of 80.46 °C), a low density of 0.09 g/cm^3^, and a relatively high compressive stress of 0.51 MPa at 10% strain.

## 1. Introduction

Climate change due to global energy resource consumption has been widely recognized as the most urgent issue for humanity in the twenty-first century. This situation is especially of great concern when considering building consumption, accounting for more than 40% of the total national energy consumption [1,2,3,4]. Developing new and efficient thermal insulation materials effectively alleviates this energy crisis [5,6,7]. 

Due to their ultra-low thermal conductivity, vacuum insulation panels and aerogels have been developed as building insulation materials [8,9,10,11]. However, vacuum insulation panels risk failure when they are slightly broken, which is especially inconvenient in the actual operation of energy-saving buildings [12,13]. In contrast, aerogel materials are optimal for thermal insulation due to their security, maneuverability, and remarkable thermal insulating properties [14,15,16]. Nevertheless, the commonly used SiO_2_ aerogels have a negative impact on the environment due to the dust produced by their inorganic components and the consumption of non-renewable resources in their useful service. Therefore, developing environmentally friendly and renewable aerogel materials is significant for alleviating the energy crisis and global climate problems.

Preparing aerogel thermal insulation materials from natural biomass sources can effectively reduce their environmental load [17,18,19,20]. Chitosan (CTS) is a derivative of chitin, the second most abundant natural polymer on the earth, and it has an extremely rich source of non-toxic and non-hazardous raw materials [21], the biocompatibility and biodegradability make it plays an important role in biomedicine [22,23,24,25]. Thus, thermal insulation materials made with CTS synthetic aerogels completely derived from natural materials would be highly compatible with energy-saving buildings. Using ambient pressure drying, Guerrero-Alburquerque et al. prepared CTS-urea aerogels with excellent performance in terms of strong machinability and thermal insulation [26]. Another study reported CTS aerogels with an ultra-high specific surface that showed significant application prospects in thermal insulation [27]. 

Although marvelous progress has been made in preparing CTS aerogels with excellent structure and properties, the massive shrinkage of these aerogels (up to ~80%) is still an issue [28]. In a previous study, the authors applied a method to inhibit the shrinkage of CTS aerogels. The study found that CTS-based aerogels shrinkage was reduced to ~20% by the binding effect of linear polymer polyvinyl alcohol (PVA) chains and CTS molecular chains [29]. Further reduction of shrinkage would be beneficial for the practical application of aerogel materials. However, no related research has been performed so far.

In this study, supramolecular interaction was introduced to inhibit CTS-based aerogels’ shrinkage. A reliable synthetic strategy was proposed to substantially restrain the uniaxial shrinkage of CTS aerogels using the physical/chemical entanglement of the nanofibrillar cellulose (NFC) chains within/outside the CTS chains as well as the supramolecular reaction of linear PVA. This strategy is based on the previous research of our team [30]. The study compared CTS-based aerogels’ shrinkage with different aspect ratios of NFC versus those without NFC. Introducing NFC enhances the skeleton structure, thereby inhibiting shrinkage, and enriches CTS-based aerogels’ microscopic morphology, thus producing a three-dimensional structure [31]. The resulting CTS/PVA/NFC hybrid aerogels (CPNAs) were characterized to explore their thermal insulation applications. The results of this work are expected to achieve further progress in overcoming the formidable challenges of shrinking biomass aerogels, rendering them a promising potential candidate for powerful insulation in the construction sector [26].

## 2. Results and Discussion

### 2.1. Proposed Reaction Process and Evidence Confirmation

**Figure 1:**
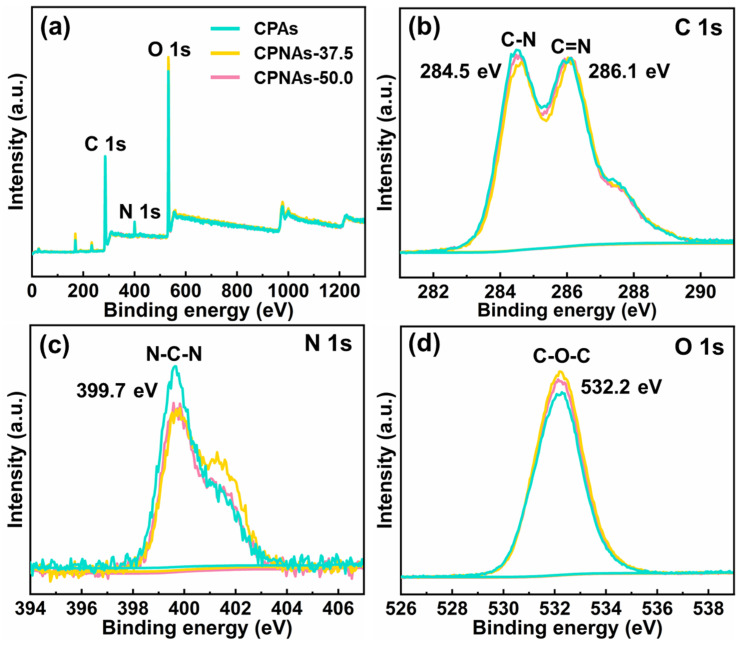
XPS spectra of CPAs and CPNAs: (**a**) full spectra, (**b**) C 1s, (**c**) N 1s, and (**d**) O 1s.

The sol-gel process is critical for forming a porous network of CPNAs. Maillard reaction is the main crosslinking mechanism of this process: activity amino (–NH_2_) groups from CTS and aldehyde (–CHO) groups from the crosslinker OPA react to produce a Schiff base (–NH=CH_2_) and then form the crosslinking bonds of N–C–N [32,33]. XPS and FTIR analysis were used to verify the chemical crosslinking reaction of the CTS aerogels. In the XPS spectrum (Figure 1), the peaks detected at binding energies of 284.5 (C 1s), 286.1 (C 1s), 399.7 (N 1s), and 532.2 (O 1s) represented CPNAs generated by C–N, C=N, N–C–N, and C–O–C, respectively [34]. XPS found no significant difference between the binding energy of the elements in the CPAs, CPNAs-37.5, and CPNAs-50.0, which indicated that the main chemical reaction in the CPNAs was the Maillard reaction, whether NFC was added or not. In brief, the network structure of principal chemical covalent bonds consisted of the above crosslinking bands in CPNAs formed by the Maillard reaction. Thus, NFC might not have participated in the chemical reaction, even while generating physical entanglement to enhance the skeleton structure.

**Figure 2:**
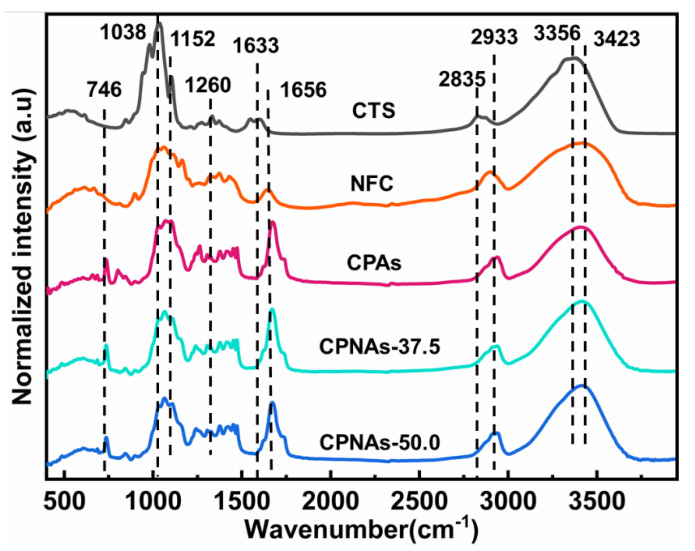
FTIR spectra of CTS, NFC, CPAs, CPNAs-37.5, and CPNAs-50.0.

The chemical structure of CPAs, NFC, CPNAs-37.5, CPNAs-50.0, and CTS, were also detected using FTIR. As shown in Figure 2, the O–H stretching bands are strong in detecting characteristic chemical groups as presented in FTIR data. Hence, the N–H bands (peaks at the range from 3356 cm^−1^ to 3423 cm^−1^) are not easily identified owing to the repeated range between O–H stretching and N–H bands in a great measure. Actually, the formed N–H bands have existed in CTS-based aerogels because of the evidence of C=N, suggesting the formation of N–H band indeed. The bands of the scissoring vibration of the –NH_2_ group appeared at 1633 cm^−1^, while the O–H and N–H stretching vibrations were detected at peaks for 3356 cm^−1^ and 3423 cm^−1^, respectively. Thus, a crucial phenomenon was found in which the absorbance strength in the CPNAs was apparently enhanced at 1656 cm^−1^ compared with that of CTS, corresponding to the stretching vibrations of C=N in nature. This result illustrated the network formation occurred due to the chemically crosslinking interaction depending upon Maillard reaction [35]. XPS and FTIR did not find a free –CHO band on OPA in the observable phase in CPNAs, implying that the absolute transformation to C–N and N–C–N bands responded accordingly.

The secondary crosslinking interaction included chemical crosslinking of the aldol condensation reaction in the sol-gel process, which was randomly intertwined with multiple angles of NFC [28]. In this interdigitated interaction process, the hydrogen bonding between –OH/–NH_2_ and –OH groups from CTS, PVA, or NFC provided a good network base frame, and introducing NFC with a nanoscale diameter, which intertwined randomly, formed a multiplicity network nanostructure. The peaks at 2835 cm^−1^ and 2933 cm^−1^ could be explained by the intermolecular and intramolecular hydrogen bonds of the CTS chain and PVA/NFC chain [36]. Finally, the possible self-crosslinking of PVA was likely due to hydroxy (namely, –OH) groups’ reaction with each other and their subsequent hydrogen-bonding integration. The crosslinking reaction decreased the two-type bands’ relatively broad adsorption between 3000 cm^−1^ and 3600 cm^−1^, which elaborated to –OH stretching. Although very subtle hydrogen bonding was detected by FTIR, concluding that this was due to the hydrogen bonding of NFC was difficult. Therefore, the probability of forming chemical bonds with the addition of NFC was weak.

### 2.2. Nanostructure Textural Evaluation

**Figure 3:**
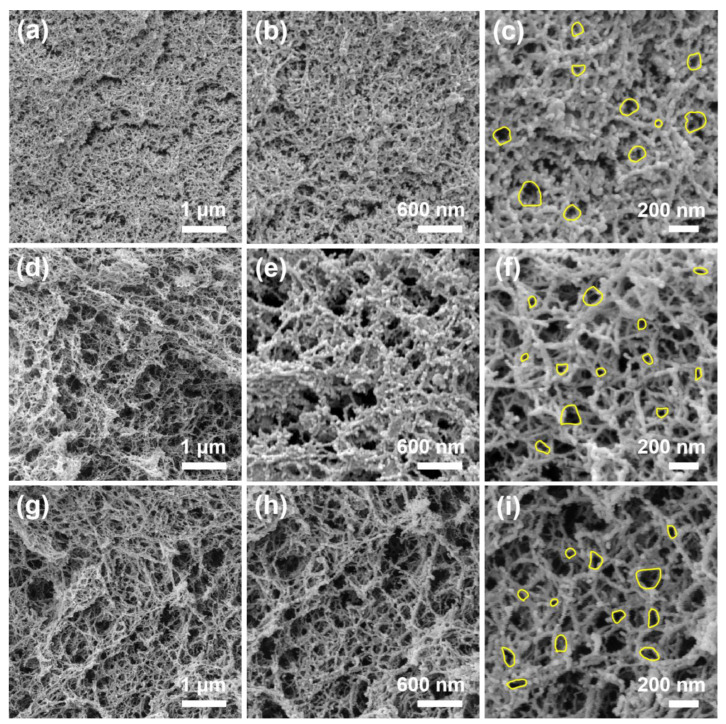
FESEM images of aerogels with various magnifications: (**a**–**c**) CPAs, (**d**–**f**) CPNAs-37.5, and (**g**–**i**) CPNAs-50.0.

The microstructural characterizations of the control and the CPNAs in Figure 3 were conducted by field emission scanning electron microscopy, which comprehensively exhibits a representative 3D interconnected network with a gradually enlarging view. Figure 3 shows FESEM images of the CPAs, CPNAs-37.5, and CPNAs-50.0. In contrast to the CPAs, the microstructure of CPNAs-37.5 and CPNAs-50.0 prepared with NFC exhibited a representative three-dimensional interconnected network like a spider web. Overall, the morphological structures of CPAs, CPNAs-37.5 and CPNAs-50.0, were composed of fibrous skeletons, which formed an interpenetrating network and were randomly intertwined to form a porous structure. However, the sufficiently tight NFC adhesion between the skeleton structure constructed a thick and stout skeleton with resistance to the influence of surface tension during the supercritical drying process, thereby producing ultra-low shrinkage CPNAs. In contrast, the microscopic appearance of the CPAs tended to be fixed, implying relatively easy shrinkage. As seen in Figure 3d–i, we have clearly found the typical pore size in FESEM images, mostly concentrating on the range within 10–60 nm and some partly focusing on 60–150 nm in pore size distribution depending on FESEM data of CPNAs-37.5, thus the impact of NFC with different aspect ratios on the morphological structures of the CPNAs was slight. Overall, introducing NFC effectively regulated and improved a sense of micro or nanoscale network structure layers through interlacement. However, the porous structure did not appear to be evenly distributed compared to the CPAs.

**Figure 4:**
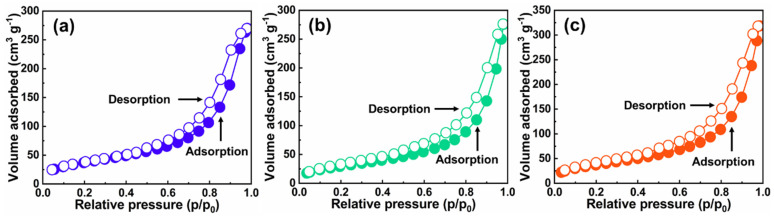
N_2_ adsorption-desorption isotherms of CPAs (**a**), CPNAs-37.5 (**b**), and CPNAs-50.0 (**c**).

**Figure 5:**
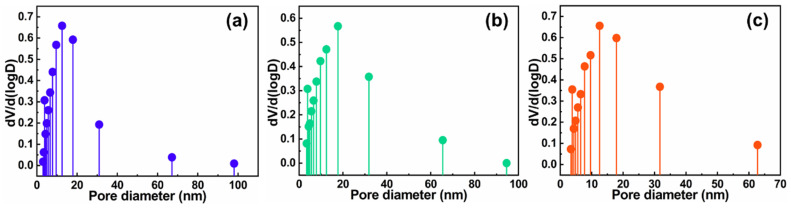
Pore size distributions of the CPAs (**a**), CPNAs-37.5 (**b**), and CPNAs-50.0 (**c**).

The results of the N_2_ adsorption-desorption test are shown in Figure 4 and Figure 5. The pore size largely existed within the mesoporous range, suggesting the formation of the nanoporous networking structure of CTS-based aerogels as-prepared. The pore size distributions of the CPAs, CPNAs-37.5 and CPNAs-50.0, were similar in a way. Although there is indeed hydrogen bonding interaction in forming the networking structure of CTS-based aerogels from FTIR data, considering the weak influence of hydrogen bonding, the hydrogen bonding here includes the interactions among the chains concerning CTS, PVA, and NFC. Therefore, we believe that the introduction of NFC has the more important effect on enhancing the skeletal structure by hydrogen bonding interaction, including the physical entanglement interaction, ultimately achieving the inhibition for the shrinkage of CTS aerogels in the drying process. 

Furthermore, although the inhibited shrinkage may have been affected by NFC, the effect of hydrogen bonding on shrinkage was minimal, as shown by FTIR [37]. The texture of the CPNAs was made up of a truss-like network, and a significant proportion of crosslinking, slice-fixed networks were present, likely due to the linear PVA chains with OPA-crosslinked CTS, as well as the bundling properties of NFC. The addition of NFC, which resulted in this truss-like texture, may have substantially controlled the resultant shrinkage due to the structural optimization [38,39]. 

### 2.3. Physical Feature Assessment

**Figure 6:**
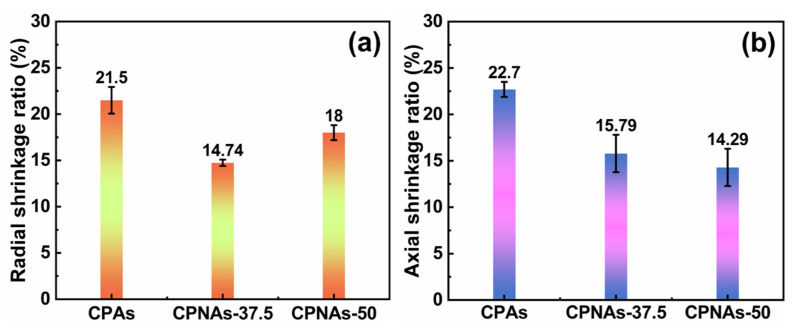
The radial (**a**) and axial (**b**) shrinkages of CPAs, CPNAs-37.5, and CPNAs-50.0 in the supercritical drying process.

The axial and radial shrinkage from the final wet gels to the aerogels is shown in Figure 6. The shrinkage of the CPAs along the radial and axial directions was 21.5% and 22.7%, respectively. The relatively low shrinkage of the CPAs can be attributed to the crosslinker (OPA) and linear PVA polymer, which bonded to the rigid benzene ring and provided the supramolecular interaction. Moreover, the inter/intramolecular physical entanglement of NFC may have further controlled the shrinkage; the radial and axial shrinkage of the CPNAs-37.5 was only 14.74% and 15.79%, respectively, which was lower than previously reported for CTS based aerogels [28] and traditional CTS aerogel, which can be as high as 50% [35]. Fortunately, the shrinkage of the CPNAs was sharply restricted within ~15% along the radial and axial directions, which was due to the trussed effect by NFC chains and linear PVA chains. In the supercritical drying process, chitosan hybrid gels with the enhanced skeletons could resist the shrinking force, namely, remaining the nanoporous networking structure of aerogels derived from the corresponding gels above, which possess accordingly the relatively large pores from FESEM and shrinkage data. Contrarily, the networking skeleton of gels becomes weaker, then the shrinkage in the supercritical drying process would be larger in the pore structure aspect.

**Figure 7:**
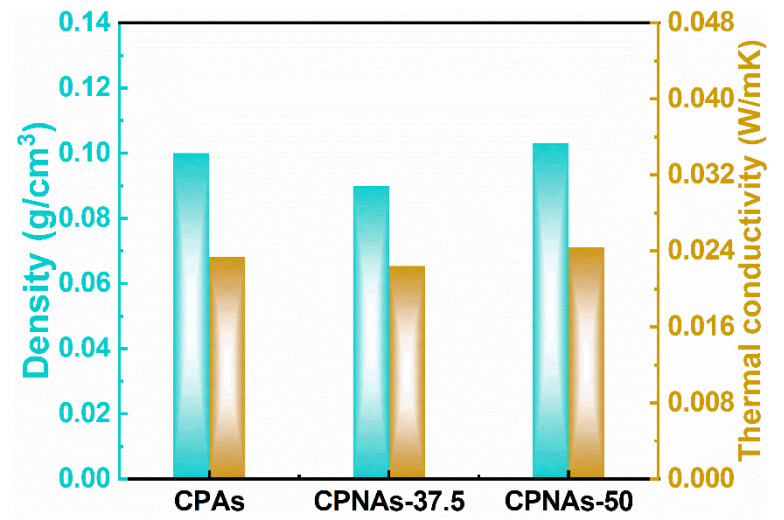
Thermal conductivity and density of CPAs, CPNAs-37.5, and CPNAs-50.0 at ambient environment.

Outstanding thermal insulation is an inherent and crucial performance of aerogel materials [40]. Figure 7 describes the density and thermal conductivity of the CPAs, CPNAs-37.5, and CPNAs-50.0. The CPNAs-37.5 were ultra-lightweight (0.09 g/cm^3^), with a low thermal conductivity of 0.0224 W/(m·K) under atmospheric pressure. The thermal conductivity of the CPNAs was close to that of silica aerogels and well below conventional insulation materials (e.g., polystyrene foam, polyurethane foam (PU), etc.) under identical conditions [41,42,43,44,45]. Thus, they have relevant application potential in energy-saving buildings.

**Figure 8:**
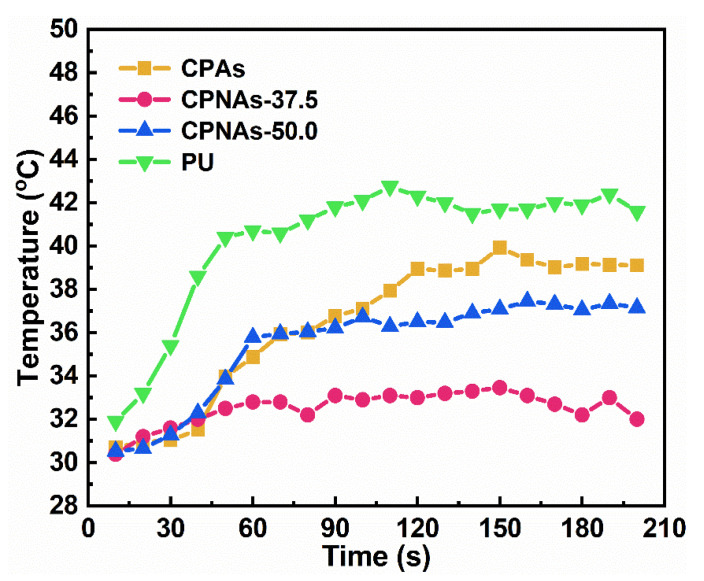
Temperature rise curves on the cold surface of CPAs, CPNAs-37.5, CPNAs-50.0, and PU.

**Figure 9:**
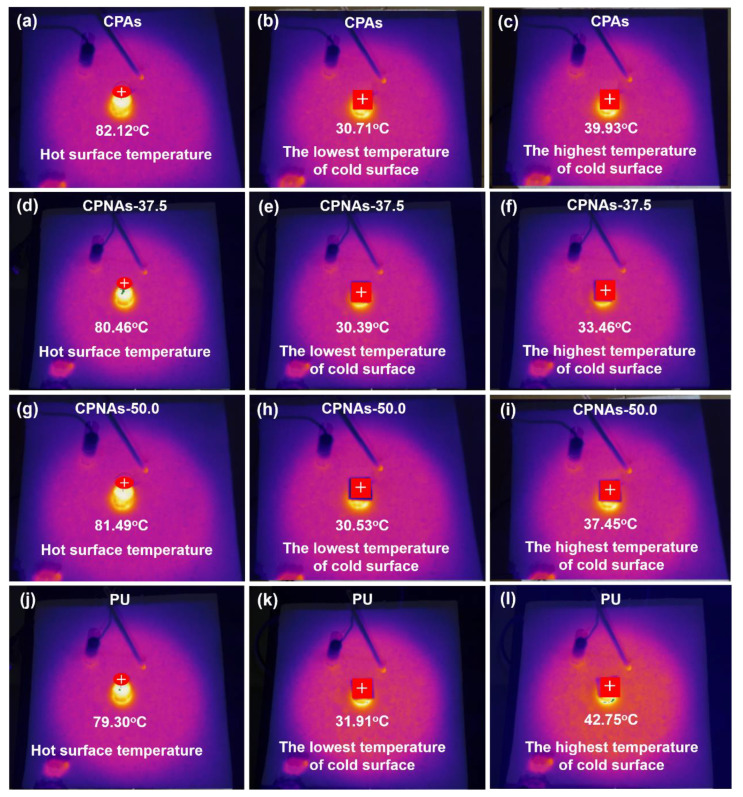
The temperature changes of the cold surface recorded by infrared thermography (left line: the original temperature of the hot surface; middle line: the starting temperature of the cold surface; right line: the highest temperature of the cold surface) of CPAs (**a**–**c**), CPNAs-37.5 (**d**–**f**), CPNAs-50 (**g**–**i**), and PU (**j**–**l**).

To further explore the effect of thermal insulation, a thermal imaging experimental device was designed to test the insulating performance. We conducted our self-made simple insulation experiments. The samples of CPAs, CPNAs-37.5, CPNAs-50.0, and PU with a thickness of 0.7 cm were placed in the port surface of a test tube. The temperature rise curves on the cold surface of the samples are shown in Figure 8. The temperature of the water in the test tube was set at 90 °C, and the true temperature can be found in Figure 9a,d,g,j. 

The CPNAs exhibited superior thermal insulation performance over PU (The density of PU is 0.0285 g/cm^3^), and the CPNAs-37.5 had a colder surface temperature at the comparative hot surface temperature. A temperature imager was used to record the temperature variation on the cold surface during the test. As shown in Figure 9, the cold surface temperature of CPAs (Figure 9b), CPNAs-37.5 (Figure 9e), CPNAs-50.0 (Figure 9h), and PU (Figure 9k) in the initial stage was 30.71 °C, 30.39 °C, 30.53 °C, and 31.91 °C, respectively.

The highest temperature of the cold surface of the CPNAs-37.5 was 33.46 °C after heating to the balanced state. This was only an increase of 3.07 °C (Figure 9e,f). The cold surface temperature of PU reached 42.75 °C, an increase of 10.84 °C (Figure 9k,l). This indicated that the CPNAs-37.5 possessed superior thermal insulation performance.

The mechanical properties of the compressive stress-strain curves of the CPAs, CPNAs-37.5 and CPNAs-50.0, are illustrated in Figure S1. All the samples could be compressed up to 75% strain without any fractures or cracks. The elastic deformation in the early strain phase was due to the linear elasticity feature at a low strain of 10% (Figure S1b–d). Compared with the CPAs, the CPNAs-37.5 and CPNAs-50.0 exhibited relatively lower compressive stress. This may have been because the addition of NFC led to higher elasticity mechanical properties in the prepared aerogel [46].

The TG and DSC curves of the CPAs (a), CPNAs-37.5 (b), and CPNAs-50.0 (c) from room temperature to 500 °C with a heating rate of 5 °C/min are shown in Figure S2. All aerogels exhibited a similar thermogravimetric process, which was roughly divided into three stages. The weight loss for the first stage was related to the evaporation of the physically absorbed water. The second stage was stabilized in the temperature range from 53 °C to 225 °C, as shown in the TG curves. Based on the acceptable thermal stability over a relatively large temperature range (53–225 °C), the CPNAs had a promising thermal insulation application for a wide temperature range. The third stage was presented by significant weight loss in the CPNAs as the temperature rose, indicating that the CPNAs had begun to decompose chemically and physically.

## 3. Conclusions

In summary, this study presented a facile strategy to regulate CTS-based aerogels’ shrinkage through the chemical crosslinking reactions, combining with the supramolecular interaction and physical entanglement with NFC at a nanoscale. The prepared CPNAs exhibited a fine nanoporous structure, low density, and ultra-low thermal conductivity (0.0224 W/m·K) greatly owing to their low shrinkage (14.74%) derived from the enhanced skeleton structures. Furthermore, the CPNAs-37.5 possessed a better thermal insulation performance than those of PU under the comparative hot surface temperature. The mechanical properties and thermal stability of CPNAs were also studied, and they presented linear elasticity behavior within the low strain range and excellent thermal stability up to 225 °C. This approach for preparing low shrinkage chitosan aerogels is expected to accelerate the production of biomass aerogels for thermal insulation in energy-saving buildings in the future.

## 4. Experimental Section

### 4.1. Materials

Low-viscosity CTS (the molecular weight: 1.8 × 10^5^–2 × 10^5^ Da; deacetylation rate: 82%), (China), acetic acid (AR), ethanol (AR), (China), o-phthalaldehyde (OPA, AR) were purchased by Aladdin Reagent Co., Ltd. (Shanghai, China), NFC (diameter: 10–300 nm) was obtained from Zhongshan NFC Bio-materials Co., Ltd. (GuangDong, China), PVA (the molecular weight: 4.4 × 10^5^ Da; alcoholysis degree: 87–89 mol%) were obtained from Sinopharm Chemical Reagent Co., Ltd. (Shanghai, China). Deionized water was utilized throughout the experiment. All reagents in this study were used without any further purification.

### 4.2. Fabrication of CPNAs

**Figure 10:**
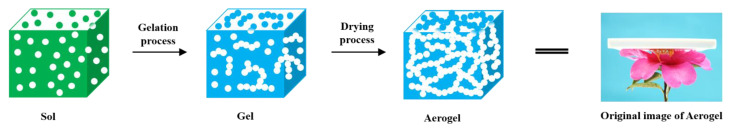
Schematic diagram of CPNAs preparation.

The typical preparation process of the CPNAs was as follows. First, 20 g of NFC (2.5 wt.%) were dropped in 90 g (4 wt.%) of PVA and stirred for 30 min to form a homogenous solid and liquid mixture. Mixtures were poured slowly into a 150 g CTS solution of 10 g/L (CTS solution of 10 g/L was prepared in the mixed solvents containing 2 wt.% acetic acid aqueous solution (40 vol.%) and ethanol (60 vol.%)) [47], and continually stirred for 30 min at the conditions of 2–10 °C to obtain a CTS/PVA/NFC solution. Second, the crosslinker of 60 g OPA (8 wt.%) was slowly added into the CTS/PVA/NFC solution at 10 °C to form the CPNAs solution. This solution was immediately poured into special molds and placed at 20 °C overnight to eliminate the bubbles caused by stirring. Then, the temperature was raised gradually during the gelation process. After gelation, the temperature rises by 10 °C for each aging treatment, aging treatment was conducted from 25 °C to 75 °C, increasing by 10 °C every 24 h. The solvent exchange was conducted several times at the same time; the fresh ethanol was added to replace the gel solvent and ultimately gain the ethanogel, which was then the supercritical CO_2_ drying at 60 °C, 18 MPa for 12 h. The obtained CPNAs are shown in Figure 10. The CPNAs with different aspect ratios of NFC were marked as CPNAs-37.5 (aspect ratio is 37.5; diameter: 10–30 nm) and CPNAs-50.0 (aspect ratio is 50.0; diameter: 300 nm). For comparison, samples without NFC were also prepared and labeled as CPAs.

### 4.3. Characterization Measurements

#### 4.3.1. Chemical Characteristic Evaluation

The chemical structure and composition of the CPNAs were detected through Mg Kα radiation using a photoelectron X-ray spectrometer (XPS; Thermo Scientific K-Alpha, Waltham, MA, USA), as well as Fourier transform infrared (FTIR) spectra (Thermo Scientific Nicolet iS5) employing KBr pellets with a sample to KBr weight ratio of 1:99 and the scanning range was 400–4000 cm^−1^.

#### 4.3.2. Surface Morphology

The morphology and microstructure of the CPNAs were analyzed by a field emission scanning electron microscope (FESEM, SU8010) with a gold coating at 10 kV.

#### 4.3.3. Nitrogen Adsorption-Desorption Test

Nitrogen adsorption-desorption isotherms were measured at 77 K using a surface area and porosimetry analyzer (Quantachrome auto-sort IQ) after the CPNAs were degassed under vacuum at 90 °C for 12 h. The Brunauer–Emmett–Teller (BET) theory calculated the specific surface area, and pore size distribution was characterized from the desorption branch using the Barret-Joyner-Halenda (BJH) model. The shrinkage and density of the CPNAs were calculated by averaging multiple measurements.

#### 4.3.4. Bulk Shrinkage and Density

The shrinkage of the sample included axial and radial shrinkage. Axial shrinkage is defined as the thickness of the sample after drying divided by the thickness before drying. In contrast, radial shrinkage is the average of the shrinkage of the length and width of the sample before and after drying. Density was obtained by dividing the weight of the sample by volume.

#### 4.3.5. Compression Measurement

Compressive stress-strain curves were recorded by a universal mechanical tester equipped with a 20-kN load cell at a 0.5 mm/min compression rate. The mechanical properties were tested under the conditions of 25 °C and humidity of 50%, and the dimensions of CPNAs-50, CPNAs-37.5 and CPAs were adopted with the size of 2 cm × 2 cm × 1 cm. 

#### 4.3.6. Thermal Conductivity Measurement

The thermal properties of the CPNAs (3–7 mg) were measured using thermogravimetry (TG, Q500, TA) and differential scanning calorimetry (DSC, Q200, TA) from 25–500 °C with a 5 °C min^−1^ heating rate in an air atmosphere. The thermal conductivity of the samples was measured using a thermal conductivity analyzer (FOX 200, TA) with dimensions of Φ20 cm at room temperature. A Fluke TiS60+ thermal imager was used to test the thermal insulation performance of the CPNAs under a heat source temperature set at 90 °C, and the true temperature could be found in Figure 9a,d,g,j. Thermal imager photos of samples were taken at a rate of every 10 s for 200 s.

## Data Availability

Data are available from the authors. Samples of the compounds are available from the authors.

## Supplementary Materials

The following are available online at https://www.mdpi.com/2310-2861/8/2/131/s1, Figure S1: Compressive stress-strain curves of CPNAs up to 75% strain (a); compressive stress-strain curves at low strain for CPAs (b), CPNAs-37.5 (c), and CPNAs-50.0 (d), Figure S2: TG and DSC curves of CPAs (a), CPNAs-37.5 (b), and CPNAs-50.0 (c).
