# Supplementary material for: Chitosan Based Aerogels with Low Shrinkage by Chemical Cross-Linking and Supramolecular Interaction
Source: Gels. 2022 Feb 18;8(2):131. doi: 10.3390/gels8020131 (PMC8924760; doi:10.3390/gels8020131)
Supplement: Supplementary file 1 [file gels-08-00131-s001.zip › gels-1585989-supplementary.pdf]

Chitosan Based Aerogels with Low Shrinkage by Chemical Cross-Linking and Supramolecular Interaction

Sizhao Zhang,[1,2,] Qi Xiao,[1] Yunyun Xiao,[1,*] Zhengquan Li,[1] Shixian Xiong,[1] Feng Ding,[1] Junpeng He[1]*

[1] Polymer Aerogels Research Center, Jiangxi University of Science and Technology, Ganzhou 341000, Jiangxi, PR China

[2] Postdoctoral Research Station on Mechanics, College of Aerospace Science and Engineering, National University of Defense Technology, Changsha 410073, Hunan, PR China

Supporting Information

** Correspondence and requests for materials should be addressed to Sizhao Zhang (E-mail: zhangsz@jxust.edu.cn).*

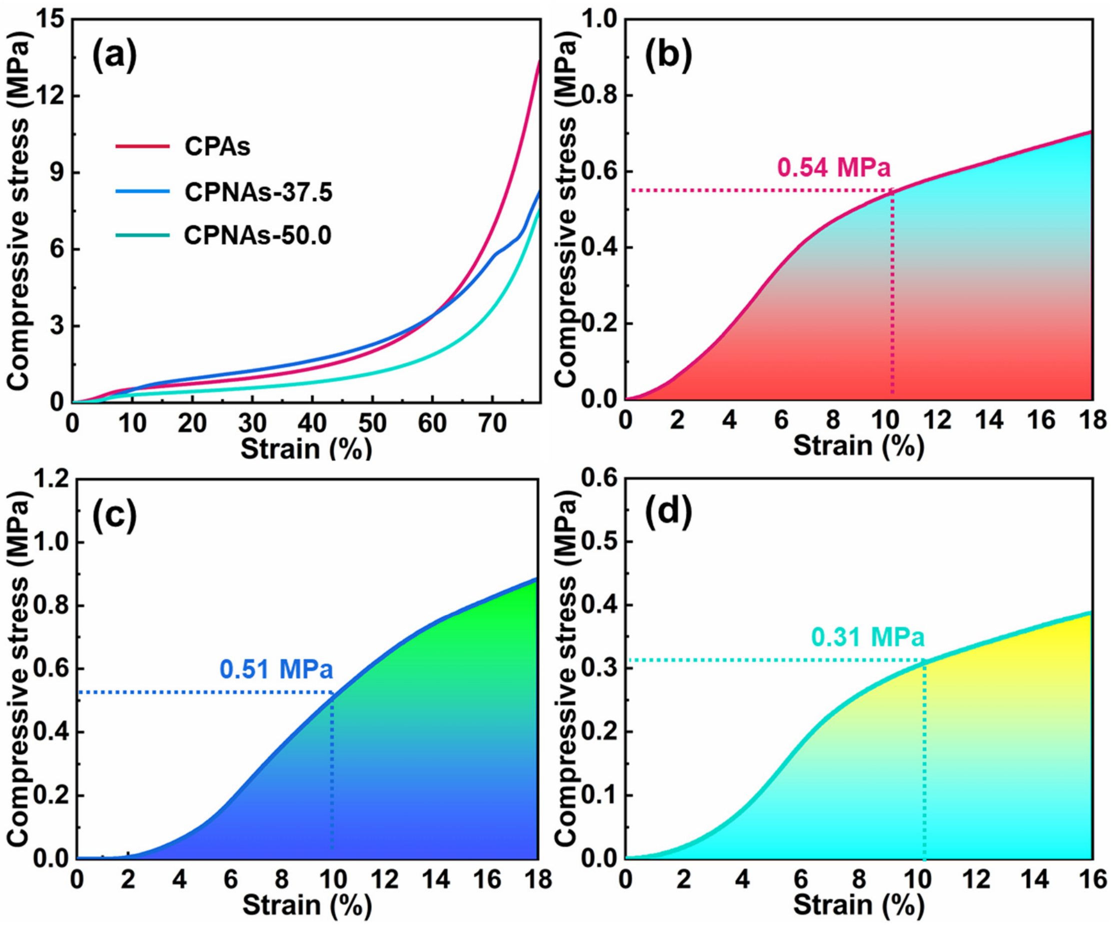

Figure S1. Compressive stress-strain curves of CPNAs up to 75% strain (a); compressive stress-strain curves at low strain for CPAs (b), CPNAs-37.5 (c), and CPNAs-50.0 (d).

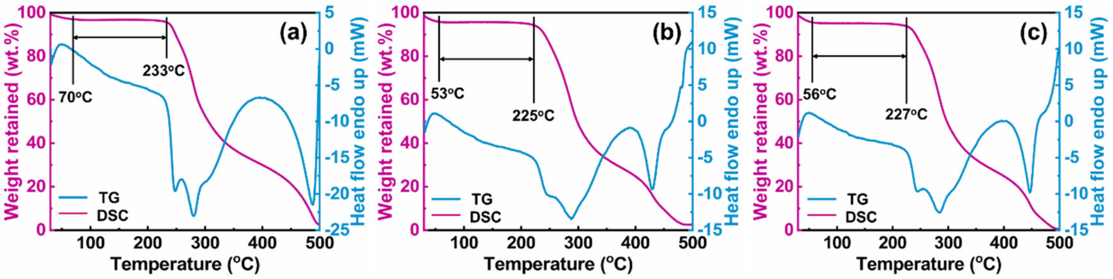

Figure S2. TG and DSC curves of CPAs (a), CPNAs-37.5 (b), and CPNAs-50.0 (c).
